# Editorial: Physical activity and a healthy diet as a medicine for obesity

**DOI:** 10.3389/fspor.2024.1477065

**Published:** 2024-08-28

**Authors:** Rizwan Ahmed Laar, Rashid Menhas, Zulkaif Ahmed Saqib, Huilin Wang

**Affiliations:** ^1^ College of Physical Education, Hubei Normal University, Huangshi, China; ^2^ Fourth Affiliated Hospital, School of Medicine, Zhejiang University, Yiwu, China; ^3^ College of Urban Transportation and Logistics, Shenzhen Technology University, Shenzhen, China; ^4^ School of Business, Hunan University of Science and Technology Xiangtan, China

**Keywords:** physical activity, healthy diet, obesity, medicine, sedentary lifestyle

**Editorial on the Research Topic**
Physical activity and a healthy diet as a medicine for obesity

Physical activity (PA) refers to any activity that consumes energy produced by skeletal muscles. The World Health Organization (WHO) narrated that PA is the first sign of community-level health (1). PA plays a significant role in physical and social development and is vital for maintaining weight gain or loss while providing cognitive, social, physical, and emotional benefits (2, 3). Apart from this, governments worldwide have highlighted the significant negative impact of sedentary lifestyles on global health-related issues.

In both developed and developing societies, non-communicable diseases (NCDs) such as obesity and chronic health conditions are typically associated with unhealthy diets and a lack of exercise (4, 5). Hypertension, smoking, high blood sugar, and obesity are among the leading causes of fatality worldwide. Obesity, in particular, is a major contributor to most of the NCDs, such as hypertension, stroke, osteoporosis, and diabetes (6). Obesity is a very complicated condition characterized by excessive body fat, with a sedentary lifestyle being one of its primary causes (7). Promoting healthy eating and PA as measures to prevent obesity (one of the deadly NCDs) is crucial for promoting an active and healthy lifestyle.

The core objective of this research topic is to reduce deaths caused by NCDs by introducing obesity prevention measures, in this case, through a healthy diet and PA. This can be achieved with the help of different tactics that motivate families and young people to engage in sports activities, such as collaborating with businesses, community organizations, and governments to fund and encourage the maintenance of fitness centers, community entertainment facilities, and parks. Apart from this, improving spatial infrastructure practices and policies can help increase access to existing community facilities and properties such as hospitals, community organizations, and schools through shared or unrestricted use policies and agreements. In addition, expanding business hours, offering scholarships, setting affordable user fees, and adopting strategies to improve the safety and security of community entertainment and park facilities (especially in resource-limited, high-crime, and geographically isolated areas), including better design features, lighting, and community security, are also essential. Finally, raising awareness among people about the negative impact of unhealthy diets, such as snacks, fast food, and sugary grains, and inadequate PA can help improve overall wellbeing.

However, PA has also been shown to cure many syndromes. Multiple studies have shown that indulging in PA can reduce the risk of metabolic syndrome, especially in smokers who are unable to quit (Kim et al., 8–10). In addition, maintaining a balanced diet and engaging in regular PA are essential for good health, as physical fitness is highly associated with body composition and the consumption of macronutrients. Obese, overweight, and underweight candidates tend to perform worse on physical fitness indicators compared to those with normal weight. Adolescents who follow the recommended levels of protein and carbohydrate intake demonstrate better physical fitness (Oukheda et al.).

The current study concluded that weight loss is proportional to the reduction of carbohydrates, junk food, and sugary diets. Reducing the intake of these types of foods is the best way to lose weight and lower the chances of obesity, especially when combined with calorie reduction and participation in PA. Likewise, the current meta-analysis shows that intermittent fasting combined with PA reduces leptin levels but does not alter adiponectin levels compared to PA alone. While reduced leptin levels may be related to weight loss, this result is inconclusive due to the inadequate number of existing studies (Kazeminasab et al., Soltani et al.).

This research promotes health through PA and a healthy diet, and it is clear that PA plays a vital role in building a healthy lifestyle. Focusing on the importance of daily PA and a healthy diet reminds us that endorsing PA is an asset of holistic and structured development. When we work together to promote the culture of PA into our daily lives, we can contribute to healthy bodies and minds that are more focused and capable of facing the challenges of life. We believe that the evidence gathered in this research topic will contribute to transforming the social environment and promoting better mental and physical health, thus laying a solid foundation for a healthier society.
